# Supplementary material for: Arp2/3 complex-driven spatial patterning of the BCR enhances immune synapse formation, BCR signaling and B cell activation
Source: eLife. 2019 Jun 3;8:e44574. doi: 10.7554/eLife.44574 (PMC6591008; doi:10.7554/eLife.44574)
Supplement: Transparent reporting form [file elife-44574-transrepform.pdf]

eLIFE
1st Floor
24 Hills Road
Cambridge CB2 1JP, UK
P 01223 855340
W elifesciences.org
T @elife

eLife's transparent reporting form

We encourage authors to provide detailed information *within their submission* to facilitate the interpretation and replication of experiments. Authors can upload supporting documentation to indicate the use of appropriate reporting guidelines for health-related research (see EQUATOR Network), life science research (see the BioSharing Information Resource), or the ARRIVE guidelines for reporting work involving animal research. Where applicable, authors should refer to any relevant reporting standards documents in this form.

If you have any questions, please consult our Journal Policies and/or contact us: editorial@elifesciences.org.

Sample-size estimation

- You should state whether an appropriate sample size was computed when the study was being designed
- You should state the statistical method of sample size computation and any required assumptions
- If no explicit power analysis was used, you should describe how you decided what sample (replicate) size (number) to use

Replicates

- You should report how often each experiment was performed
- You should include a definition of biological versus technical replication
- The data obtained should be provided and sufficient information should be provided to indicate the number of independent biological and/or technical replicates
- If you encountered any outliers, you should describe how these were handled
- Criteria for exclusion/inclusion of data should be clearly stated
- High-throughput sequence data should be uploaded before submission, with a private link for reviewers provided (these are available from both GEO and ArrayExpress)

Please outline where this information can be found within the submission (e.g., sections or figure legends), or explain why this information doesn't apply to your submission:

• *We address these questions together on the next page.*

eLife Sciences Publications, Ltd is a limited liability non-profit non-stock corporation incorporated in the State of Delaware, USA, with company number 5030732, and is registered in the UK with company number FC030576 and branch number BR015634 at the address 1st Floor, 24 Hills Road, Cambridge CB2 1JP | August 2014

1. Sample size estimation: We did not perform power calculations to determine the sample size in advance as we relied on post-hoc statistical analysis (p-values determined using a paired 2-tail t-test, the Mann Whitney U test, or the Kolmogorov-Smirnov test, as appropriate) to reveal whether or not the distributions of values for two samples being compared to each other were significantly different. When these analyses revealed that the distributions of values for the two samples were not significantly different, we did not pursue whether these were perhaps false negatives as the differences between the median values for the two samples were often quite small. In this case, we did carry out some post-hoc power calculations. In general, these revealed that we would have had to analyze an impractical number of samples (e.g. single cell imaging of hundreds or thousands of cells) to rule out the possibility that we were missing small (but perhaps not biologically significant) differences between the two samples.

2. Replicates: All key experiments that are depicted in the figures were performed at least 3 times (i.e. 3 independent experiments = 3 biological replicates). The number of independent experiments (biological replicates), as well as the sample size (e.g. # of cells per time point within one experiment), are reported in the figure legends.

a) Single cell imaging and videos of real-time imaging: For each figure panel or video, multiple independent experiments were performed and for each condition multiple cells were imaged in each experiment.

b) Quantification of single cell fluorescence microscopy data (signaling, Ag gathering data): multiple independent experiments were performed in which >20 cells (often 40-120 cells) were analyzed for each point. Dot plots showing the values for individual cells from a single representative experiment are shown in order to depict the cell-cell variability. Median values and interquartile values are shown on these graphs. To demonstrate reproducibility, data from multiple experiments are compiled by showing the mean $\pm$ SEM for the median values from multiple independent experiments.

• **Outlier testing:** For these experiments, outlier testing was performed as described in the Statistical Analysis subsection in the Materials and Methods. Outliers were identified using Robust Regression and Outlier Removal (ROUT) in GraphPad Prism with Q set to 1%, as described by Motulsky and Brown (BMC Bioinformatics, 7:123, 2006)

c) Immunoblotting: Multiple independent experiments were performed. Band intensities were quantified from representative experiments. Data from multiple experiments were compiled by comparing relative band intensities. Images of the full blots are shown in Figure 9-Figure supplement 4.

d) Single-particle tracking: In a single experiment, 900-5300 individual receptors (from 30-100 cells) were tracked for each condition. Each experiment was performed at least 3 times and a single representative experiment is shown.

e) Flow cytometry: For each figure panel, multiple experiments were performed. Gating strategies and sample calculations are shown in Figure 9-Figure supplement 1 and Figure 9-Figure supplement 2. For each sample run on the flow cytometer, >10,000 events were collected

eLife Sciences Publications, Ltd is a limited liability non-profit non-stock corporation incorporated in the State of Delaware, USA, with company number 5030732, and is registered in the UK with company number FC030576 and branch number BR015634 at the address 1st Floor, 24 Hills Road, Cambridge CB2 1JP | August 2014

Statistical reporting

- Statistical analysis methods should be described and justified
- Raw data should be presented in figures whenever informative to do so (typically when N per group is less than 10)
- For each experiment, you should identify the statistical tests used, exact values of N, definitions of center, methods of multiple test correction, and dispersion and precision measures (e.g., mean, median, SD, SEM, confidence intervals; and, for the major substantive results, a measure of effect size (e.g., Pearson's r, Cohen's d)
- Report exact p-values wherever possible alongside the summary statistics and 95% confidence intervals. These should be reported for all key questions and not only when the p-value is less than 0.05.

Please outline where this information can be found within the submission (e.g., sections or figure legends), or explain why this information doesn't apply to your submission:

The statistical tests used are described in the Statistical Analysis subsection in the Materials and Methods section. Two-tailed paired t-tests were used to compare mean values for matched sets of samples. The Mann-Whitney U test was used to compare ranks in samples with many cells and high variability (e.g. dot plots for fluorescence values). The Kolmogorov-Smirnov test was used to compare cumulative frequency distributions and FRAP curves.

For each data panel, N values are reported in the figure legends, along with the statistical test used. p-values are shown on the figures or in the figure legends.

(For large datasets, or papers with a very large number of statistical tests, you may upload a single table file with tests, Ns, etc., with reference to sections in the manuscript.)

Group allocation

- Indicate how samples were allocated into experimental groups (in the case of clinical studies, please specify allocation to treatment method); if randomization was used, please also state if restricted randomization was applied
- Indicate if masking was used during group allocation, data collection and/or data analysis

Please outline where this information can be found within the submission (e.g., sections or figure legends), or explain why this information doesn't apply to your submission:

Not applicable.

Additional data files ("source data")

- We encourage you to upload relevant additional data files, such as numerical data that are represented as a graph in a figure, or as a summary table
- Where provided, these should be in the most useful format, and they can be uploaded as "Source data" files linked to a main figure or table
- Include model definition files including the full list of parameters used
- Include code used for data analysis (e.g., R, MatLab)
- Avoid stating that data files are "available upon request"

Please indicate the figures or tables for which source data files have been provided:

The automated image analysis scripts have been uploaded to public websites. The links are in the Methods section.
